# Animal performance, rumen microbiota, and fermentation in growing camel calves fed alfalfa hay, Atriplex, or their mixture

**DOI:** 10.1186/s12917-025-05109-9

**Published:** 2025-11-15

**Authors:** Alaa Emara Rabee, Ahmed A. Aman, Ahmed R. Askar

**Affiliations:** Animal and Poultry Nutrition Department, Desert Research Center, Ministry of Agriculture and Land Reclamation, Cairo, Egypt

**Keywords:** Camels calves, Forage type, Atriplex, Rumen microbiota, Fermentation, Growth

## Abstract

**Background:**

Insights into the effect of *Atriplex* on the performance of growing camels can support *Atriplex* as an alternative feed resource for sustainable agriculture. This study evaluated the effect of replacing alfalfa hay with *Atriplex nummularia* hay on rumen microbiota and fermentation, and the growth performance of camel calves.

Twenty-four one-year camels were allocated into three groups (*n* = 8) to feed on one of three forage types for 120 days: concentrate feed mixture (CFM) at 0.9% of body weight (BW) and ad libitum Alfalfa hay (HH); CFM and ad libitum mixture of *Atriplex* hay and Alfalfa (1:1) (MM); and CFM and ad libitum* Atriplex* (AA).

**Results:**

Bacterial community was dominated by phyla Bacteroidota and Firmicutes. Atriplex inclusion increased the relative abundance of genera *Christensenellaceae R-7 group*, and *Acetitomaculum*. while Atriplex declined genera *Anaeroplasma* and *Fibrobacteres as well as* rumen methanogens that were dominated by genus *Methanobrevibacter*. Total volatile fatty acids (VFAs) was similar between camel groups; and camels fed *Atriplex* had low rumen ammonia and acetic acid, and higher propionic and butyric acids (*P* < 0.05). Feed intake was similar, and growth rates were 655.00, 540, and 466 g/head/d for groups HH, MM, and AA, respectively, with a significant difference (*P* < 0.05).

**Conclusions:**

*Atriplex* altered the rumen microbiota and fermentation with a slight negative effect on animal growth. Therefore, *Atriplex* can replace alfalfa hay partially in the feeding of camel calves in the presence of CFM.

## Background

Halophyte plants are an integral part of the sustainability of the agricultural sector in arid and semi-arid regions as they can grow using saline water and soils, where other plants can hardly grow [1]. Furthermore, these plants can be used in animal feeding during the prolonged dry seasons [2]. Compared to high-quality forages (Alfalfa), halophyte plants have higher productivity under saline conditions [3]. However, halophytes, such as *Atriplex*, have similar protein and crude fiber and higher salt and antinutritional factors than those of high-quality forages [3, 4], which negatively affected the palatability and rumen fermentation of these plants [3]. Therefore, it is necessary to select the well-adapted animal species to ensure optimum utilization of halophyte plants.

Dromedary camels are well adapted to the harsh conditions in the arid zones due to their unique behavioral, physiological, and nutritional adaptive characteristics that enable them to withstand extreme adverse environmental stresses such as drought and feeding shortage [5]. Camel production is challenged by the fluctuation of feed and water during the dry season [2]. Camels can utilize poor-quality fodders such as thorny bushes and halophytes that are avoided by other animals [6]. Higher salt requirements of dromedary camels compared to other livestock species contribute to the adaptation of camels to feeding on halophytes [2].

Understanding the interaction between the quality and quantity of available halophyte plants and rumen microbiota and fermentation would improve the efficient use of these plants in animal feeding [5]. Forage type is the main determinant of the rumen microbiota and fermentation, and animal performance [6]. Rabee et al. [7] indicated that tannin-rich plants, such as Atriplex, incubated in camel rumen, were colonized with higher proportions of members of phyla Bacteroidetes and Firmicutes, such as *Prevotella*, *RC9_gut_group*, and *Butyrivibrio*, while other genera, such as *Fibrobacteres* and *Anaerovibrio*, declined. Furthermore, **s**upplementation of dairy calves with gallic acid (subclass of tannins) increased the rumen propionic and butyric acid production as well as the relative abundance of rumen *Prevotella_1*, *Saccharofermentans*, and *Prevotellaceae_UCG-001* [8]. However, Dadvar et al. [5] noticed declines in rumen cellulolytic enzymes, ammonia, and volatile fatty acids (VFA) in camels fed *Atriplex*.

Previous studies on camels [2, 4] indicated that camels fed *Atriplex* showed higher feed intake and similar growth performance to camels fed clover hay. Feeding sheep and goat on *Atriplex* solely affected their performance negatively; therefore, including readily available carbohydrates is recommended to improve the utilization of saltbush in the rumen [1].

However, no prior studies linked the performance with rumen fermentation and microbiota in camel calves fed Atriplex. The insight into the relation between rumen microbiota, growth performance, and saltbushes could contribute to including the saltbushes in camel fattening, which contributes to food security in the marginal areas [2, 7]. Therefore, this study aims to investigate the effects of replacing high-quality forage (alfalfa hay) with saltbushes (*Atriplex* hay) on the rumen microbiota and fermentation, feed intake, and growth performance of dromedary camel calves.

## Material and methods

### Ethics

This study was approved and conducted following the regulations and guidelines of the Institutional Animal Care and Use Committee of the Desert Research Center, Cairo, Egypt (approval number: AN05042021). All protocols, including sample collection, followed the ARRIVE 2.0 guidelines (https://arriveguidelines.org). The study does not include clinical trials and animal euthanasia. The camel calves used in this study are the offspring of the camel herd in El-Shalateen Research Station, Desert Research Center, El-Shalatein, Red Sea Governorate, Egypt. All animals were released to the camel herd after the end of the experiment (after 120 days).

### Animals and diets

The study was conducted at the farm of the National Campaign for the Promotion of Camel Productivity, Wadi-Hodien, El-Shalateen Research Station, which belongs to Desert Research Center, El-Shalatein, Red Sea Governorate, Egypt. Twenty-four one-year-old male camel calves (*Camelus dromedarius*), with an average body weight of 164.4 ± 7.65 kg, were used in the current experiment. All animals were assessed for health issues, and all of them were healthy and free of health issues and diseases.

Animals were housed individually in half-shaded, sandy floor pens (8*12 m^2^) with free access to drinking water. All the animals received a concentrate feed mixture at 0.9% as DM (dry matter) of their live body weight (BW) [2, 4], and the roughage was offered to the animals ad libitum.

The calves were divided into three groups (*n* = 8) to receive one of three forages ad libitum: Alfalfa hay solely (HH); a mixture of Alfalfa hay and *Atriplex* hay (50:50) (MM); or *Atriplex* hay solely (AA). Animals received the concentrate supplement individually and forage twice a day at 08:00 and 14:00 h. The animals were adapted to the experimental diets for 15 days before the start of the study. The tender part of the *Atriplex* (*Atriplex nummularia*) growing in the Wadi-Hodeen area, El-Shalateen Research Station, was collected and sun-dried. Alfalfa hay was bought from the commercial market. The alfalfa hay and *Atriplex* hay were mixed thoroughly to be introduced to the second group (MM). The proximate analysis of concentrate, alfalfa hay, and *Atriplex* is presented in Table 1. The study lasted four months, and the camel calves were weighed weekly using a 1000 kg digital weight scale, and the corresponding concentrate and roughage were adjusted accordingly.

**Table 1:** Chemical composition of concentrate feed mixture, alfalfa hay, and Atriplex hay

**Ingredients**	^*^Concentrate feed	**Alfalfa hay**	**Atriplex hay**
Dry matter**,** g/kg	943	935	861
Organic matter, g/kg	887	881	748
Crude protein, g/kg	141	127	118
Neutral detergent fiber, g/kg	387	510	510
Acid detergent fiber, g/kg	198	398	367
Phytochemicals			
Phenols, %	0.05	0.09	0.90
Flavonoids, %	0.39	0.15	0.95
Tannins, %	0.17	0.24	2.10

^*^The concentrate consisted of 55% corn, 15% soybean meal, 10% cottonseed meal, 15% wheat bran, 2.5% limestone, 1.5% salt, 0.5% sodium bicarbonate, 0.1% yeast, 0.1% antitoxins, and 0.3% premix

### Rumen samples

By the end of the experiment, rumen content from animals was collected 2–3 h after the morning feeding using stomach tubing. The first 100 mL of rumen content was discarded to avoid the saliva. The rumen content was filtered through two cheesecloth layers. The rumen fluids were used to determine rumen VFA and ammonia, as well as microbial DNA extraction. To measure ammonia and VFA, 1 mL of rumen sample was acidified with 200 μL of meta-phosphoric acid 25% (w/v). Then, the samples were centrifuged at 13,000 rpm for 15 min, and the supernatant was used for VFA and ammonia measurements. Rumen ammonia (NH_3_-N) was measured calorimetrically using ammonia assay kits (Biodiagnostic, Cairo, Egypt). VFAs were measured by a gas chromatography system (TRACE 1300, Thermo Fisher Scientific, Waltham, United States) using a capillary column (TR-FFAP 30 m × 0.53 mmL D × 0.5 μm) and nitrogen was used as the carrier gas. The calibration was done using a standard with known concentrations of VFAs. The methane production was predicted using the concentration of propionic acid, Methane yield = 316/propionate + 4.4 [9].

### DNA extraction and PCR amplification

Microbial DNA was isolated from 0.5 mL of rumen samples. The sample was centrifuged at 13,000 rpm for 15 min, and the precipitated pellets were used in DNA extraction using the QIAamp DNA Stool Mini Kit (Qiagen, Hilden, Germany) according to the manufacturer’s guidelines. The quality and quantity of extracted DNA were checked via gel electrophoresis and a Nanodrop spectrophotometer 2000 (Thermo Scientific, Massachusetts, United States). Rumen bacteria was investigated by amplification of the V4 region on 16S rDNA using 515 F (5-GTGYCAGCMGCCGCGGTAA-3) and 926R (5-CCGYCAATTYMTTTRAGTTT-3) primers [6] based on the following PCR amplification conditions: 94 °C for 3 min; 35 cycles of 94 °C for 45 s, 50 °C for 60 s, and 72 °C for 90 s; and 72 °C for 10 min. Finally, PCR amplicons were purified and sequenced using the Illumina MiSeq system (Illumina, California, United States).

### Bioinformatics analysis

The generated paired-end raw sequence reads were analyzed using the DADA2 pipeline (version 1.11.3) using the R platform (version 3.5.2) [10]. The generated fastq files of sequence reads were demultiplexed, and their quality was checked based on the quality scores. Only the samples with a quality score > 30 were kept for the following analyses. Consequently, six samples (two per group) were discarded from the analysis pipeline. The sequences were filtered, trimmed, and dereplicated, followed by merging read 1 and read 2 together to get denoised sequences. The chimeras were removed from the denoised sequences to generate Amplicon Sequence Variants (ASVs). Taxonomic assignment of ASVs was conducted using a combination of the functions assignTaxonomy and assignSpecies, and was compared using the SILVA reference database (version 138). The analysis pipeline was set to remove all the sequences of protozoa and mitochondria and to keep the bacterial and archaeal sequences. For all samples, the alpha diversity indices (observed ASVs, Chao1, Shannon, and Inverse Simpson) were calculated to analyze richness and evenness differences between the different groups. Beta diversity was determined as principal coordinate analysis (PCoA) and visualized using the phyloseq and ggplot R-packages. The source codes of the DADA2 pipeline are available at: https://github.com/benjjneb/dada2. The raw sequence reads are available at https://www.ncbi.nlm.nih.gov/sra/PRJNA1291447..

### Chemical composition

Dried feeds were ground and analyzed according to the method of AOAC [11] to measure dry matter (DM, method 930.15), crude protein (CP, method 954.01), ash (method 942.05), and ether extract (EE, method 920.39). Neutral detergent fiber (NDF) and acid detergent fiber (ADF) were measured using ANKOM Technology (ANKOM Technology, New York, United States) [12]. The estimation of total phenolics, total flavonoids, and total tannins in *Atriplex,* alfalfa hay, and CFM was described in Rabee et al. [13].

### Statistical analyses

Data of the relative abundances of microbial groups were tested for normality and homogeneity by the Shapiro–Wilk test, and non-normal variables were then arcsine transformed. The effect of forage type on the differences in feed intake, growth performance, rumen fermentation parameters, microbial diversity, and the relative abundances of rumen bacteria was examined by the post hoc Duncan test in one-way ANOVA at *P* < 0.05. Principal component analysis (PCA) and Bray–Curtis Permutational Multivariate Analysis of Variance (PERMANOVA) were conducted to examine the effect of forage type on animal performance and rumen microbiota using the data feed intake, growth performance, rumen fermentation parameters, microbial diversity, and the relative abundances of rumen bacteria**.** The statistical analyses were performed using SPSS v. 20.0 software package [14].

## Results

### Chemical composition of forages

The chemical analysis of alfalfa and *Atriplex* hay (Table 1) indicated that *Atriplex* has lower OM and CP compared to Alfalfa hay. In addition, *Atriplex* has higher phenols, flavonoids, and tannins than alfalfa hay.

### Diversity of the bacterial community

**Fig. 1:**
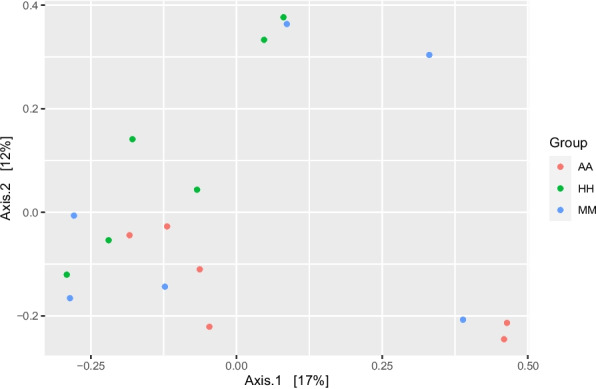
Principal coordinates analysis (PCoA) of the bacterial community was performed based on Bray–Curtis dissimilarity. The analyses were conducted between three camel groups: green circles for the camel calves fed Alfalfa hay (HH), blue circles for camel calves fed a mixture of Alfalfa hay and Atriplex hay (MM), and red circles for camels fed Atriplex hay (AA)

The sequencing of 16S rDNA amplicons resulted in a total of 843253 high-quality sequence reads with a mean of 52,703 ± 10,322 sequence reads per sample. The roughage type did not affect the alpha diversity indices (observed ASVs, Chao1, Shannon, and Inverse Simpson) of the bacterial community (Table 2). Beta diversity of the bacterial community was determined and visualized as principal coordinate analysis (PCoA), which showed that groups HH and AA were separated from each other, while group MM was not separated from the other groups (Fig. 1).

**Table 2:** Alpha diversity indices of rumen microbial community of camel calves fed Atriplex instead of alfalfa at different levels in the diet

	Concentrate feed at 0.9% of body weight			SEM	*p*-value
	HH	MM	AA		
	Mean	Mean	Mean		
Animal number	6	6	6	-	-
Observed ASVs	633.4	741.25	684.00	74.96	*p* > 0.05
Chao1	633.4	741.25	684.00	74.96	*p* > 0.05
Shannon	4.75	4.88	4.90	0.12	*p* > 0.05
Invers Simpsone	36.08	52.34	47.02	8.18	*p* > 0.05

*SEM* Standard error of means, *HH* Camels fed Alfalfa hay, *MM* Camels fed a mixture of Atriplex hay and Alfalfa, *AA* Camels fed Atriplex hay

### Composition of microbial community

The rumen microbiota of camel calves was classified into 12 bacterial phyla. In addition, rumen methanogens were detected and were affiliated to phylum Euryarchaeota (0.08%). The bacterial community was dominated by phylum Bacteroidota (61.69%), Firmicutes (34.78%), and Spirochaetota (1.99%). In addition, bacterial phyla that represented less than 1% of the bacterial community, including Actinobacteriota (0.38%), Chloroflexi (0.085%), Cyanobacteria (0.03%), Desulfobacterota (0.21%), Elusimicrobiota (0.04%), Fibrobacterota (0.046%), Planctomycetota (0.42%), Proteobacteria (0.14%), and Verrucomicrobiota (0.07%) (Table 3). Roughage type affected the relative abundance of bacterial groups. Phylum Actinobacteriota, which was affiliated with genus *Bifidobacterium*, showed its higher relative abundance in camel group AA that fed on 100% *Atriplex* compared with group HH that fed alfalfa, and group MM that fed a combination of alfalfa and *Atriplex* (*P* < 0.05) (Tables 3 and 4).

**Table 3:** The relative abundance (%) of bacterial phyla and rumen archaea in the rumen of camel calves fed Atriplex instead of alfalfa at different levels in the diet

	Concentrate feed at 0.9% of body weight			SEM	*p*-value
	HH	MM	AA		
	Mean	Mean	Mean		
Animal number	6	6	6	-	-
Actinobacteriota	0.08^a^	0.05^a^	1.27^b^	0.17	*P* < 0.05
Bacteroidota	61.92	65.16	57.00	1.80	*p* > 0.05
Chloroflexi	0.07	0.08	0.11	0.01	*p* > 0.05
Cyanobacteria	0.02	0.04	0.03	0.005	*p* > 0.05
Desulfobacterota	0.15	0.20	0.32	0.065	*p* > 0.05
Elusimicrobiota	0.04^a^	0.02^a^	0.08^b^	0.008	*P* < 0.05
Fibrobacterota	0.04^a^	0.08^a^	0.01^b^	0.009	*P* < 0.05
Firmicutes	35.79	29.82	39.48	2.04	*p* > 0.05
Planctomycetota	0.33^a^	0.44^ab^	0.63^b^	0.06	*P* < 0.05
Proteobacteria	0.043	0.06	0.41	0.10	*p* > 0.05
Spirochaetota	1.33^b^	1.55^b^	0.55^a^	0.15	*P* < 0.05
Verrucomicrobiota	0.04	0.08	0.10	0.02	*p* > 0.05
Euryarchaeota	0.09^b^	0.10^b^	0.05^a^	0.008	*P* < 0.05

*SEM* Standard error of means, *HH* Camels fed Alfalfa hay, *MM* Camels fed a mixture of Atriplex hay and Alfalfa, *AA* Camels fed Atriplex hay

^a,b^Means having different superscripts within the same row differed significantly (*P* < 0.05)

**Table 4:** The relative abundance (%) of bacterial and archaeal genera in the rumen of camel calves fed atriplex instead of alfalfa at different levels in the diet

	Concentrate feed at 0.9% of body weight			SEM	*p*-value
	HH	MM	AA		
	Mean	Mean	Mean		
Animal number	6	6	6	-	-
P: Actinobacteriota; F: Bifidobacteriaceae					
G: Bifidobacterium	0.05^a^	0.05^a^	1.36^b^	0.19	*P* < 0.05
P: Bacteroidota; F: Prevotellaceae					
F: Prevotellaceae	38.01	39.68	31.48	1.53	*p* > 0.05
G: Prevotella	34.93	35.65	28.90	1.50	*p* > 0.05
G: Prevotellaceae UCG-003	0.93^a^	1.51^b^	0.72^a^	0.13	*P* < 0.05
G: Alloprevotella	0.020^a^	0.06^b^	0.003^a^	0.008	*P* < 0.05
P: Bacteroidota; F: Rikenellaceae					
F: Rikenellaceae	4.93	6.49	5.56	0.43	*p* > 0.05
G: Rikenellaceae RC9 gut group	4.52	5.83	5.04	0.38	*p* > 0.05
F: PeH15	0.08	0.05	0.09	0.02	*p* > 0.05
F: F082	15.97	14.66	14.50	0.57	*p* > 0.05
F: Muribaculaceae	2.36	2.01	2.45	0.42	*p* > 0.05
F: Bacteroidales BS11 gut group	0.27	0.35	0.27	0.03	*p* > 0.05
F: Bacteroidales UCG-001	0.17^a^	0.38^b^	0.10^a^	0.04	*P* < 0.05
F: p-251-o5	0.45	0.94	1.59	0.26	*p* > 0.05
F: Bacteroidales RF16 group	0.23^b^	0.28^b^	0.06^a^	0.03	*P* < 0.05
F: Desulfovibrionaceae; G: Desulfovibrio	0.12	0.19	0.29	0.06	*p* > 0.05
P: Elusimicrobiota; F: Endomicrobiaceae					
G: Endomicrobium	0.03^a^	0.02^a^	0.08^b^	0.009	*P* < 0.05
P: Firmicutes; F: Ruminococcaceae					
F: Ruminococcaceae	2.60	1.71	3.19	0.37	*p* > 0.05
G: Ruminococcus	0.15	0.28	0.13	0.04	*p* > 0.05
G: Ruminococcaceae_unclassified	2.44	1.42	3.06	0.36	*p* > 0.05
P: Firmicutes; F: Lachnospiraceae					
F: Lachnospiraceae	4.98	4.10	4.80	0.50	*p* > 0.05
G: Butyrivibrio	1.33	0.91	0.24	0.22	*p* > 0.05
G: Acetitomaculum	0.31^a^	0.51^a^	1.36^b^	0.12	*P* < 0.05
G: Moryella	0.06^a^	0.15^a^	0.37^b^	0.05	*P* < 0.05
G: Lachnospiraceae UCG-009	0.02	00	00	00	ND
G: Syntrophococcus	00	0.02	0.03	00	ND
P: Firmicutes; F: Christensenellaceae					
F: Christensenellaceae	3.59^a^	3.43^a^	6.27^b^	0.4	*P* < 0.05
G: Christensenellaceae R-7 group	3.09^a^	2.68^a^	5.49^b^	0.36	*P* < 0.05
P: Firmicutes; F: Oscillospiraceae					
F: Oscillospiraceae	5.31	4.91	5.20	0.40	*p* > 0.05
G: Colidextribacter	0.05	0.03	00	00	ND
F: Erysipelotrichaceae	2.1	2.56	0.8	0.48	*p* > 0.05
F: Erysipelatoclostridiaceae; G: Sharpea	2.35	1.78	0.76	0.42	*p* > 0.05
P: Firmicutes; F: Anaerovoracaceae					
F: Anaerovoracaceae	0.57	0.53	1.05	0.10	*p* > 0.05
G: Anaerovorax	0.13^a^	0.21^a^	0.33^b^	0.02	*P* < 0.05
P: Firmicutes; F: Hungateiclostridiaceae					
F: Hungateiclostridiaceae	0.45	0.45	0.47	0.04	*p* > 0.05
G: Saccharofermentans	0.42	0.38	0.43	0.04	*p* > 0.05
G: Ruminiclostridium	0.01^a^	0.05^b^	0.02^a^	0.006	*P* < 0.05
F: Acholeplasmataceae	0.28^b^	0.16^ab^	0.08^a^	0.03	*P* < 0.05
F: Acholeplasmataceae; G: Anaeroplasma	0.25^b^	0.13^ab^	0.06^a^	0.03	*P* < 0.05
F: Acidaminococcaceae; G: Succiniclasticum	0.07	0.08	0.18	0.03	*p* > 0.05
F: Selenomonadaceae	0.07	0.035	0.19	0.05	*p* > 0.05
F: Anaerofustaceae; G: Anaerofustis	0.02	0.01	0.04	0.005	*p* > 0.05
F: Peptococcaceae	0.02	0.01	0.05	0.007	*p* > 0.05
F: Monoglobaceae; G: Monoglobus	0.02	0.03	0.013	0.003	*p* > 0.05
P: Planctomycetota; F: Pirellulaceae					
G: p-1088-a5 gut group	0.29^a^	0.33^a^	0.6^b^	0.04	*P* < 0.05
P: Spirochaetota;F: Spirochaetaceae					
Sphaerochaeta	0.65^a^	1.25^b^	0.26^a^	0.14	*P* < 0.05
Treponema	0.48	0.33	0.19	0.06	*p* > 0.05
Archaea; P: Euryarchaeota					
G: Methanobrevibacter	0.09^b^	0.10^b^	0.05^a^	0.008	*P* < 0.05

*SEM* Standard error of means, *HH* Camels fed Alfalfa hay, *MM* Camels fed a mixture of Atriplex hay and Alfalfa, *AA* Camels fed Atriplex hay, *ND* Non-determined

^a,b^Means that having different superscripts within the same row differed significantly (*P* < 0.05)

Phylum Bacteroidota dominated the bacterial community and was affiliated with families Prevotellaceae, Rikenellaceae, PeH15, F082, Muribaculaceae, Bacteroidales BS11 gut group, Bacteroidales UCG-001, p-251-o5, Bacteroidales RF16 group, and Desulfovibrionaceae. Families Bacteroidales UCG-001 and Bacteroidales RF16 group had lower relative abundance in the AA group, compared to group HH and MM, respectively (*P* < 0.05). On the genus level, this phylum was dominated by *Prevotella* and *Rikenellaceae RC9 gut group*. Candidate genera *Prevotellaceae UCG-003* and *Alloprevotella* within phylum Bacteroidota showed their higher relative abundance in the MM group compared with HH and AA (Table 4) (*P* < 0.05). Phylum Elusimicrobiota was affiliated with genus *Endomicrobium*, which was higher in group AA than in the HH and MM groups (Tables 3 and 4) (*P* < 0.05). Phylum Fibrobacterota showed its lowest proportion in the AA group (Table 3) (*P* < 0.05).

Phylum Firmicutes, the second largest phylum in the bacterial community, was classified into families Ruminococcaceae, Lachnospiraceae, Christensenellaceae, Oscillospiraceae, Erysipelotrichaceae, Anaerovoracaceae, Hungateiclostridiaceae, Acholeplasmataceae, Acidaminococcaceae, Selenomonadaceae, Anaerofustaceae, Peptococcaceae, and Monoglobaceae (Table 4).

Some genera within Phylum Firmicutes, including *Acetitomaculum*, *Moryella*, *Christensenellaceae R-7* group, and *Anaerovorax*, were higher in the AA group compared to the HH and MM groups(*P* < 0.05) (Table 4). In addition, genus *Ruminiclostridium* was higher in the MM group compared to the HH and AA groups, and the genus *Anaeroplasma*, which was decreased by the inclusion of *Atriplex* (*P* < 0.05) (Table 4). Phylum Planctomycetota was dominated by p-1088-a5 gut group and was higher in *Atriplex*-supplemented groups (MM and AA) (Tables 3 and 4) (*P* < 0.05). Phylum Spirochaetota was dominated by *Sphaerochaeta*, which was enriched in group MM compared to the HH and AA groups (Tables 3 and 4) (*P* < 0.05). Rumen methanogens (phylum Euryarchaeota) were affiliated with genus *Methanobrevibacter*, which had lower relative abundance in *Atriplex-*fed groups (Tables 3 and 4) (*P* < 0.05).

### Rumen fermentation

Roughage type affected the rumen fermentation parameters (Table 5). Replacing the alfalfa hay with *Atriplex* decreased the concentration of rumen ammonia (*P* < 0.05). Moreover, higher acetic acid was observed in group HH and MM, compared to that of the AA group (*P* < 0.05) (Table 5). Group AA showed higher propionic, butyric, and isovaleric acids compared to the HH and MM groups. Moreover, higher isobutyric and valeric acids were observed in group HH (Table 5) (*P* < 0.05). Moreover, the difference in total VFA was not significant (Table 5). The lowest predicted methane was observed in group AA compared with HH and MM (*P* < 0.05) (Table 5).

**Table 5:** Rumen fermentation parameters of camel calves fed Atriplex instead of alfalfa at different levels in the diet

	Concentrate feed at 0.9% of body weight			SEM	*p*-value
	HH	MM	AA		
	Mean	Mean	Mean		
Animal number	8	8	8	-	-
Ammonia, mg/dl	13.19^b^	6.39^ab^	4.72^a^	1.12	*P* < 0.05
Acetic, mM	59.71^b^	59.15^b^	46.40^a^	2.38	*P* < 0.05
Propionic, mM	22.82^a^	24.25^a^	28.34^b^	0.79	*P* < 0.05
Isobutyric, mM	2.18^b^	0.94^a^	1.90^b^	0.20	*P* < 0.05
Butyric, mM	25.80^a^	25.44^a^	31.60^b^	1.09	*P* < 0.05
Isovaleric, mM	5.77^b^	3.22^a^	7.99^c^	0.55	*P* < 0.05
Valeric, mM	11.09^b^	8.82^a^	9.71^a^	0.33	*P* < 0.05
Total VFA, mM	127.39	121.84	125.96	2.48	*p* > 0.05
Predicted methane, g/kg DMI	17.98^b^	17.04^b^	15.27^a^	0.38	*P* < 0.05

*SEM* Standard error of means, *HH* Camels fed Alfalfa hay, *MM* Camels fed a mixture of Atriplex hay and Alfalfa, *AA* Camels fed Atriplex hay, *mM* Millimolar

^a,b,c^ Means having different superscripts within the same row differed significantly (*P* < 0.05)

### Feed intake and growth performance

The initial body weight of camel calves was similar between camel groups (*p* > 0.05). Roughage type affected the average daily gain (ADG), which was slightly declined in groups fed *Atriplex* (MM and AA) (*P* < 0.05) (Table 6). Camels fed 50% (MM) and 100% (AA) *Atriplex* achieved 82% (540 g/d) and 71% (466 g/d) of the growth rate of group HH fed alfalfa hay (655 g/d). Feed intake expressed as g/kg BW^0.75^ was similar between experimental groups (*p* > 0.05). Furthermore, a higher feed conversion ratio (FCR) was observed in group AA compared to HH and MM (*P* < 0.05) (Table 6).

**Table 6:** Growth performance and feed intake of growing camel calves fed Atriplex instead of Alfalfa at different levels in the diet

Items	Concentrate feed at 0.9% of body weight			SEM	*p*-value
	HH	MM	AA		
	Mean	Mean	Mean		
Fattening period, days	120	120	120		
Animal number	8	8	8	-	-
Initial body weight, kg	167.00	163.00	163.00	7.64	*p* > 0.05
Final body weight, kg	236.00	218.00	213.00	8.55	*p* > 0.05
Body weight changes, kg	68.40^b^	54.83^a^	49.60^a^	2.28	*P* < 0.05
Average daily gain, g/day	655.00^b^	540.00^a^	466.00^a^	24.91	*P* < 0.05
Dry matter intake, g/kg BW^0.75^					
Concentrate	5.78^a^	6.56^ab^	7.50^b^	0.25	*P* < 0.05
Hay	38.68	19.06	0	ND	ND
*Atriplex*	0	18.75	37.46	ND	ND
Total Roughage	38.68	37.80	37.46	0.39	*p* > 0.05
Total	70.58	69.23	68.86	0.70	*p* > 0.05
Feed conversion ratio	5.78^a^	6.56^ab^	7.50^b^	0.25	*P* < 0.05

*SEM* Standard error of means, *ND* Non-determined, *HH* Camels fed Alfalfa hay, *MM* camels fed a mixture of Atriplex hay and Alfalfa, *AA* Camels fed Atriplex hay

^a,b^Means having different superscripts within the same row differed significantly (*P* < 0.05)

### Principal component analysis (PCA)and Bray–Curtis Permutational Multivariate Analysis of Variance (PERMANOVA)

**Fig. 2:**
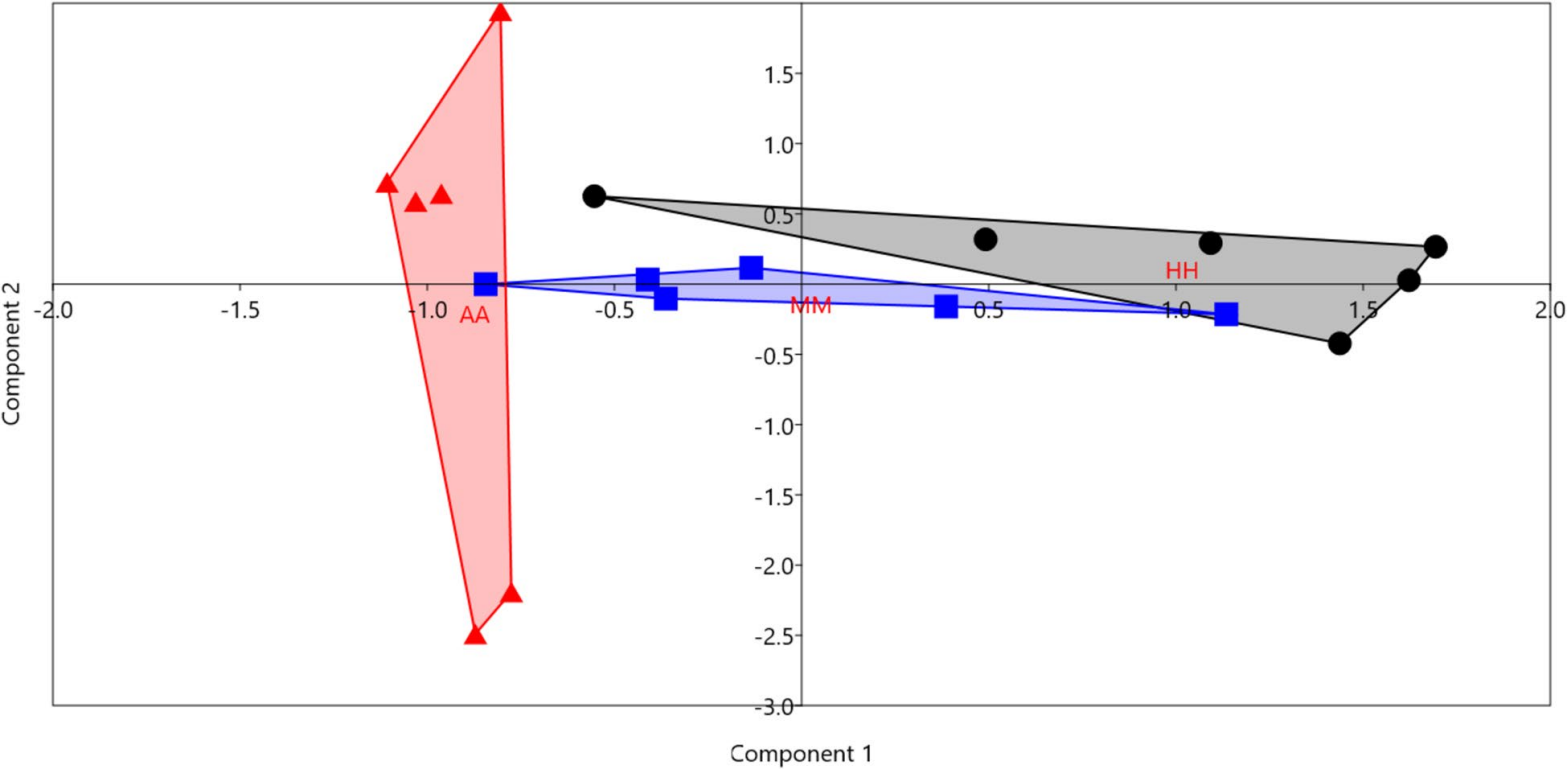
Principal component analysis (PCA). PCA analysis was conducted between experimental groups using the results of growth performance, rumen fermentation parameters, microbial diversity, and the relative abundances of rumen bacteria. The black dots are camel calves fed Alfalfa hay (HH), blue squares are for camel calves fed a mixture of Alfalfa hay and Atriplex hay (MM), and red triangles are for camels fed Atriplex hay (AA)

PCA analysis (Fig. 2) showed that the samples were separated based on the forage type, and this finding was supported by a significant difference (*p* = 0.0053) obtained by the PERMANOVA test. The clustering in PCA was driven by ADG, acetic, propionic, total VFA, methane, and the relative abundance of *Christensenellaceae R-7 group*, and *Rikenellaceae RC9 gut group.*

## Discussion

Understanding the interaction between the chemistry of forage plants, rumen microbiota, and fermentation, and performance in camel calves can help design suitable feeding strategies that use halophyte plants as alternatives to conventional feeds. The proximate analysis of *Atriplex* and alfalfa hay in this study followed the range of previous studies [2, 15]. Tannin content of *Atriplex* was lower than the value indicated in Dadvar et al. [5] and higher than that in Mahipala et al. [16]. In addition to phenols, tannins, and flavonoids, *Atriplex* contains different antinutritional factors, such as saponins, oxalates, alkaloids, and resins [7]. The tannin and phenols content of alfalfa hay was much lower than *Atriplex*, and the values indicated by Seyedin et al. [17]. Secondary metabolites in the halophyte plants can modify the rumen microbiota and fermentation [6].

### Rumen microbiota and rumen fermentation

The majority of the bacterial community belonged to phyla Bacteroidota and Firmicutes, which agrees with the previous study on tanniniferous plant incubated in camel rumen [6, 7] and *Lotus corniculatus* [18]. The members of Bacteroidota are specialized in the degradation of polysaccharides such as hemicellulose, cellulose, and pectin [7, 18]. Inclusion of the *Atriplex* in the camel diet did not affect the relative abundance of phylum Bacteroidota or its dominant family, Prevotellaceae, which indicates that this phylum resists the secondary metabolites of saltbushes (*Atriplex*) and has a role in the performance of animals [7]. This speculation is supported by the higher prevalence of genus *Alloprevotella* in *Atriplex*-fed camels (MM and AA) compared to camels fed alfalfa hay (HH). A similar finding was obtained in lambs fed mimosa condensed tannin [19]. *Alloprevotella* produces acetate and succinic acid and can enhance rumen fermentation and animal health [20]. Candidate genera *Prevotellaceae UCG-003* and *Bacteroidales UCG-001* can resist tannins and have potential roles in fiber degradation [21, 22], which demonstrates their higher proportions in *Atriplex*-fed groups. In contrast, the family Bacteroidales RF16 group declined in group AA fed 100% Atriplex. This family has a potential role in fiber degradation [23].

Some members of the phylum Firmicutes, such as family Ruminococcaceae and genera *Acetitomaculum*, *Moryella*, *Ruminiclostridium*, *Christensenellaceae R-7 group,* and *Anaerovorax,* were higher in *Atriplex-*fed groups. These finding indicates that these bacteria can resist tannins [6, 7]. In addition, these bacteria can degrade fiber and produce VFA such as acetic and butyric acids using H_2_, which reduces the methane production and improves feed efficiency [13, 24–27]. Tannin-resistant bacteria contribute to the feed efficiency and ability of camels to utilize thorny bushes, halophytes [6, 7]. In contrast, some fibrolytic bacteria were declined by inclusion of the *Atriplex*, such as *Anaeroplasma* [28].

Moreover, some minor phyla were increased in *Atriplex*-fed calves, including Elusimicrobiota, Actinobacteriota, and Planctomycetota. The members of these phyla can resist tannins [6, 7, 13, 29] and ferment glucose and complex carbohydrates to acetic and lactic acids, and it was associated with higher feed efficiency in calves [29–32].

Phyla Fibrobacterota and Spirochaetota were declined in *Atriplex*-fed groups. The members of these phyla are sensitive to tannins [7, 13, 19]. Moreover, these bacteria are involved in the metabolism of complex carbohydrates such as pectin and cellulose and produce acetic acid [6, 33, 34], which is a negative point of feeding on saltbushes.

Rumen methanogens were affiliated mainly with the genus *Methanobrevibacter*, which declined in AA groups. The decline of the *Methanobrevibacter* could be attributed to the direct effect of secondary metabolites (tannins, phenols, saponins) that have antimicrobial effects on the methanogens or the protozoa that provide rumen methanogens with hydrogen for methane production [5, 13, 35]. Another explanation for the decline in the rumen methanogens is the low availability of acetate and hydrogen [13, 36]. Previous studies [6, 36] indicated that tannin-rich plants decreased rumen methanogens, methane production, and protozoa count.

The changes in forage type affected the rumen fermentation parameters, due to the changes in the rumen microbial community [5, 13]. The decline in the fiber-degrading and acetate-producing bacteria, such as *Fibrobacteres* and Sphaerochaeta, might have decreased the fiber digestibility and the acetate production [7, 33, 34], which explains the lower acetic acid in the *Atriplex*-fed camels [5]. Previous studies on camels [5, 37], sheep and goat [38] reported that the inclusion of halophytes in the diets decreased the cellulolytic enzymes and digestibility. The higher production of propionate, as in the group AA, consumes the hydrogen from the rumen environment, which decreases the availability of hydrogen for methane production, as the hydrogen is the main substrate for rumen methanogens to produce methane [13]. This explanation is supported by lower predicted methane in the AA group. Higher propionic and butyric acids in the AA group could be attributed to the higher butyric and propionic-producing bacteria, such as *Prevotella*, *Anaerovorax*, and *Moryella,* which tolerate the secondary metabolites in *Atriplex* [13, 25, 27]. Similar findings were obtained in claves supplemented with gallic acid [8]. The total VFA was not affected in camels fed Atriplex, which agrees with previous studies on camels and lambs fed *Atriplex* and camelthorn [5, 37, 39]. The decline in the rumen ammonia was also reported in camels fed *Atriplex* [5]. Abdullah et al. [40] noticed a decline in the rumen ammonia in lambs fed *Atriplex* hay. Lower rumen ammonia could be attributed to the lowered degradation of peptides and deamination of amino acids in the rumen due to the presence of tannins in the *Atriplex* [4, 5, 39].

### Feed intake and growth performance

The values of feed intake were within the ranges indicated in growing camels fed different forages [2, 4]. Including the *Atriplex* in camel diets did not affect the feed intake. In contrast, previous studies on camels [2, 4] reported increased feed intake when Alfalfa and Rice straw were replaced by *Atriplex*. Growth performance was slightly declined due to the inclusion of *Atriplex* in animal diets; however, the values of ADG in the current study were higher than those of camels fed alfalfa hay and *Atriplex* [2, 4]. In contrast, Farid et al. [2] and Abdel-Wahed [4] reported improved growth rate due to replacing clover hay and rice straw with *Atriplex*, and they attributed the increase of ADG in camels fed *Atriplex* to the ad libitum feeding of fresh chopped *Atriplex*; while *Atriplex*, in the current study, was offered dried. The decline in growth performance could be attributed to the decrease in digestibility as reported on growing camels fed *Atriplex* or camelthorn [37, 41]. Similar findings were obtained on sheep and goats fed *Atriplex* [38, 40]. Furthermore, higher tannins and phenols hinder the absorption of nutrients by animals [42], which justifies the decline in the growth of *Atriplex*-fed groups, which have approximately similar rumen VFA to group HH fed Alfalfa hay.

## Conclusion

Inclusion the *Atriplex* in camel calves’ diet increased some bacterial genera that have important roles in rumen fermentation, such as *Christensenellaceae R-7 group*, while other bacteria and rumen methanogens were declined. These changes decreased methane and ammonia production, while total VFA was similar in the experimental groups. Consequently, the growth rate in camels fed a mixture of Atriplex and Alfalfa hay was slightly decreased. Therefore, *Atriplex* is recommended in the feeding of camel calves in the presence of high-quality forages and concentrate feed mixture. Future studies are recommended to study more inclusion levels of *Atriplex* with restricted feeding to study the effect of *Atriplex* inclusion on digestibility, carcass characteristics, and meat quality.

## Data Availability

The raw sequence reads are available at: https://www.ncbi.nlm.nih.gov/sra/PRJNA1291447.

## Abbreviations

ADG: Average daily gain
ASVs: Amplicon Sequence Variants
BW: Body weight
CFM: Concentrate feed mixture
DM: Dry matter
PERMANOVA: Bray-Curtis Permutational Multivariate Analysis of Variance
PCA: Principal component analysis
PCoA: Principal coordinate analysis
VFA: Volatile fatty acids
